# Association of *SLCO1B1* gene variants with angiotensin-converting enzyme inhibitor-induced cough in a Pakistani hypertensive cohort

**DOI:** 10.3389/fphar.2024.1441251

**Published:** 2024-09-04

**Authors:** Arooj Fatima Sheikh, Nayla Munawar, Rukhsana Nawaz, Hizbullah Khan, Mehwish Rafique, Faryal Jahan, Sagheer Ahmed

**Affiliations:** ^1^ Shifa College of Pharmaceutical Sciences, Shifa Tameer-e-Millat University, Islamabad, Pakistan; ^2^ Department of Chemistry, College of Science, United Arab Emirates University, Al Ain, United Arab Emirates; ^3^ Department of Clinical Psychology, College of Medicine and Health Sciences, United Arab Emirates University, Al Ain, United Arab Emirates; ^4^ The Center for Microbes, Development and Health, CAS Key Laboratory of Molecular Virology and Immunology, Shanghai Institute of Immunity and Infection, Chinese Academy of Sciences, Shanghai, China; ^5^ University of Chinese Academy of Sciences, Beijing, China; ^6^ Shifa International Hospital, Islamabad, Pakistan

**Keywords:** ACE inhibitors, dry cough, single nucleotide polymorphisms, adverse effects, SLCO1B1

## Abstract

**Background:**

Angiotensin-converting enzyme inhibitors (ACEIs) are prescribed for individuals with high cardiovascular (CV) risk; however, persistent cough limits the use of ACEIs in a large number of patients. The current study aimed to identify the genetic variants in the *SLCO1B1* gene that might be associated with ACEI-related cough in a Pakistani hypertensive population.

**Methods:**

A prospective cohort study was conducted at a tertiary care hospital in Pakistan. A total of 74 patients who had been treated with ACEIs were recruited through a convenient sampling method. The study was approved by the Institutional Review Board & Ethics Committee of the Shifa International Hospital, Islamabad. Patients provided 2 ml of blood for sequencing after signing informed consent. Partial gene sequencing of *SLCO1B1* was carried out to find single nucleotide polymorphisms (SNPs) and haplotypes.

**Results:**

It was found, through a structured questionnaire, that thirty-eight (38) patients experienced cough within 2 weeks of ACEI administration and were considered as a case group (cough), and thirty-six (36) patients were considered as a control group (no cough). The incidence of cough was 51%. We found six different SNPs and 9 haplotypes in the partial gene sequences of *SLCO1B1.* Haplotype H4 was associated significantly with cough after adjusting for sex and smoking status. Other SNPs and haplotypes were not significantly associated with ACE-Is-induced cough.

**Conclusion:**

These findings emphasize the significance of *SLCO1B1* genetic variants, specifically H4, as a potential predictor of ACEI-induced cough. It could be included in clinical practice as a possible risk factor for ACEI-induced cough once confirmed in larger clinical trials with bigger sample sizes. The replication of these findings in larger and more diverse populations is likely to contribute to the therapeutic use of ACEIs by predicting ACEI-induced cough.

## Introduction

Hypertension is a serious risk factor for stroke, myocardial infarction, coronary heart disease, myocardial infarction, and end-stage renal disease (James et al., 2014). Between 1990 and 2019, its global prevalence has doubled, from 650 million to 1.3 billion people (WHO, 2023). The prevalence of hypertension in the Pakistani population is high, 16% in the rural and 21.6% in the urban population in individuals over 15 years. The majority of hypertensive patients in Pakistan are unaware of their hypertension. The total number of people with hypertension in Pakistan is around 5.5 million men and 5.3 million women. Only a tiny fraction (less than 3%) use proper medications to have controlled hypertension (Kearney et al., 2005; Soomro et al., 2013; Ahmed et al., 2008). In the Pakistani population over 45 years, around one-third are suffering from hypertension (Ghaffar et al., 2004).

Several remedies both natural and synthetic, have been employed to maintain normal blood pressure (Ali et al., 2023; Hussain et al., 2010),. Angiotensin-converting enzyme inhibitors (ACEIs) were recommended by Joint National Committee VII (JNC-7) as one of the safest medications for the treatment of hypertensive patients with comorbidities including chronic renal disease, diabetes, or congestive heart failure because ACE-Is do not adversely affect heart and kidneys (Khalil et al., 2001). Due to this safety profile, the clinical application of ACEIs to treat hypertension has dramatically grown. Globally, between 20 and 30 million patients take these medications to control hypertension (Messerli et al., 2018). The worldwide market size of the ACEIs is around 3,456.27 USD Million. In Pakistan, ACEIs are prescribed to most of the patients admitted to hospitals for the treatment of MI (Alam et al., 2024).

Compared to other cardiovascular (CV) drugs, ACEIs have no more harmful side effects. However, the most frequent adverse effect related to the use of ACEIs is a chronic cough (Nikpoor et al., 2005). Cough can start right away after the first dose or it can take weeks or months to appear after the start of the therapy. This bothersome dry cough frequently becomes severe enough to necessitate stopping the medication because reducing the dose of the drug will not stop it as this effect is dose-independent. Data on the prevalence of cough are conflicting (Punzi, 1993). Retrospective or post-marketing studies have shown it to be as low as 1-2 percent, while controlled trials have reported it to be as high as 37–39 percent. Coughing can range in intensity from an irritating, dry tickle to a chronic hacking that is accompanied by sleeplessness or vomiting. The frequency of ACEI-related cough varies by the patient’s sex, smoking habits, race, and other conditions. Therefore, ACEI-related cough is more common in women, non-smokers, Asian people, and congestive heart failure (CHF) patients (Gibson, 1989; Israili and Hall, 1992).

The cause of ACEI-induced cough is still not fully understood. It has been suggested that the pathogenic mechanism of ACEI-induced cough may involve the accumulation of chemicals that ACE typically metabolizes, such as bradykinin and substance P, in the airways and the subsequent stimulation of vagal afferent neurons that service the cough reflex (Sheikh et al., 2022). The onset of cough, however, might be the result of a more intricate series of circumstances than first thought. Bradykinin has been demonstrated to activate proinflammatory pathways in the body by increasing the production of arachidonic acid metabolites including prostaglandins and nitric oxide, which in turn promotes coughing (Trifilieff et al., 1993).

The *SLCO1B1* gene codes for the solute carrier organic anion transporter family member 1B1 (*SLCO1B1*). *SLCO1B1,* also known as *OATP1B1*, is located in liver cells and aids in the hepatic clearance of several medications, including ACE inhibitors (Pasanen et al., 2007). Since this gene is involved in the uptake of ACE inhibitors from the blood into hepatocytes, its expression as well as function are critical to the efficacy and adverse effects of ACE inhibitors. This fact makes *SLCO1B1* a promising gene to study for investigating ACE inhibitor-induced cough. There are more than 190 single nucleotide polymorphisms (SNPs) known to exist in the *SLCO1B1* gene (Liu et al., 2006). According to earlier studies, among the 190 SNPs identified, the two most prevalent ones (521T>C, rs4149056, and 597C>T, rs2291075) are responsible for variations in the pharmacokinetics (PK) and pharmacodynamics (PD) of the *SLCO1B1* substrate (Oshiro et al., 2010). However, no research on the relationship between *SLCO1B1* mutations and ACEI-induced cough has been done in Pakistan. Due to the extensive usage of ACEIs, physicians must be able to anticipate each patient’s response to the medication to reduce the frequency of coughing. Therefore, the purpose of this study is to determine whether the incidence of cough in a Pakistani hypertensive cohort treated with ACEIs is associated with genetic variation, specifically SNPs and haplotypes in *SLCO1B1*.

## Methods and materials

### Study approval, participants & data collection

A prospective cohort study was conducted with patients attending the clinic of their physicians at a tertiary care hospital, Shifa International Hospital (SIH) and Shifa Plus Pharmacy, in Pakistan. The study approval was taken from the institutional review board (IRB) and Ethics Committee (EC) of SIH, Islamabad, Pakistan, under the letter (IRB# 058–22). Patients were enrolled prospectively via convenient sampling. The sample size was calculated by taking a 95% confidence level, 0.10 absolute precision, and anticipated prevalence of ACE inhibitor-induced cough, 46% as per the literature review, a sample size of 79 was calculated by using the WHO sample size calculator. After obtaining informed consent standardized per the WHO guidelines, patients were requested to participate through a structured questionnaire. Their baseline information comprised age, gender, body weight, height, and smoking status. ACEI-related characteristics include type, duration of ACEI usage, and occurrence of cough. The duration of the cough was also recorded. Only patients who met all of the inclusion criteria were included in this study. Patients with respiratory tract infection (RTI) and/or pneumonia were excluded. Eligible subjects prescribed with ACEIs were interviewed via phone call after 2 weeks of therapy.

### DNA extraction and sequencing

All study participants gave 2 milliliters (mL) of venous blood in a sterile tube containing etheylenediaminetetraacetic acid (EDTA). Genomic DNA was extracted from leukocytes per the protocol described previously (Hussain et al., 2023) and kept at −20°C. A NanoDrop spectrophotometer was used to measure the DNA concentration.

Targeted sequencing of the *SLCO1B1* gene was performed by SeqStudio™ (Applied Biosystems USA). We selected a hypervariable region of the *SLCO1B1* gene which is a known hotspot of variation. This approach of selected hypervariable hotspots has previously yielded good results (Ahmed et al., 2022). The amplified PCR products were cycle sequenced with a BDT v.3.1 master mix as per the manufacturer’s instructions, for both the forward and reverse primers. BigDye Xterminator™ (Thermofisher Scientific USA) kit was used for the purification of the cycle-sequenced product as per the manufacturer’s instructions. Samples were loaded for analysis, and run parameters were set according to the kits used. A medium run was selected for electrophorese. The nucleotide sequence data of all the individuals generated in this study are available from Genebank under the accession numbers PP387529- PP387595.

### Sequencing data analysis

To ensure accuracy, we carried out a trace quality check on the DNA sequence reads using the Staden Package (Staden et al., 1999) and Finch TV v1.4.0 (http://www.geospiza.com/Products/finchtv.shtml). The high-quality sequence reads were assembled using Lasergene v.7.1 packages (DNASTAR Inc.USA) and Bio-Edit software (http://www.mbio.ncsu.edu/BioEdit/bioedit.html) as mentioned previously (Khokhar et al., 2021).

### Identification of SNPs & haplotypes in *SLCO1B1* gene

The high-quality filtered sequence data was compared to the reference genome of the GRCh38.p14 in the Ensemble database (Howe et al., 2021) using Blast. The sequences of the whole cohort were aligned using the MUSCLE program (Edgar, 2004) in MEGA-X (Kumar et al., 2018). The SLCO1B1 gene’s in-house alignment region (located at 12:21178627–21179056) in the Ensemble database was successfully analyzed. The analysis covered partial exon 6, whole exon 7, whole intron 7, and partial intron 8. The haplotypes were generated using the DnaSP v6.12 software package (Rozas et al., 2017). This process involved converting the DnaSP v6.12 output into the Arlequin project file format (.arp). The number of polymorphic sites and haplotype frequencies was computed using Arlequin v3.5 (Excoffier et al., 2005). Within Arlequin v3.5, a new project was initiated specifying all the samples of the population (e.g., coughers vs. non-coughers) and the molecular data type (DNA sequences). In the Arlequin, the data parameters were set at the haplotypic level, and locus level, and searched to capture the shared haplotypes among all samples. The software was used to infer haplotypes and generated a unique haplotype list that was present in the samples, along with their frequencies.

### Statistical analysis

All data were analyzed using IBM Statistical Package for Social Sciences (SPSS) version 23.0. Cronbach alpha was used to measure the reliability of the questionnaire. Mean was calculated for continuous data and frequencies were calculated for categorical data. Chi-square was also applied to examine the association of *SLCO1B1* polymorphisms with ACEI-induced cough. Multivariate logistic regression analysis was used for adjusting sex, and smoking status. *P*-value ≤ 0.05 was taken as significant.

## Results

### Basic demographic and clinical parameters

Basic demographic and clinical parameters are mentioned in Table 1. There was no statistically significant difference between the coughers and non-coughers at *p* < 0.05. Initially, 90 patients were recruited for the study. Of these, 10 patients (11.1%) were excluded because of catching pneumonia, and 6 patients (6.6%) were excluded for other reasons. Finally, the investigated cohort included 74 essential hypertensive patients receiving ACEIs, including 39 (52%) males and 35 (47%) females. The mean age and BMI were 51.28 years and 24.5 kg/m^2^, respectively. The mean SBP and DBP of subjects at the baseline were 125- and 80-mm Hg, respectively. Twenty-three percent (23%) of patients were smokers, fifty-five percent (55%) had a history of hypertension, and the largest ethnic group in our study was Punjabi, which makes up about 71% of the study sample, followed by Pashtuns (21%), Kashmiris (4%), and Sindhis (2.7%). Ramipril was the most frequently prescribed ACEI (60%), followed by Captopril (20%), enalapril (10.8%), and lisinopril (8%). The number of patients with cough once a week was significantly more (*p* < 0.001) than those with twice or more cough episodes per week (Table 1).

**TABLE 1:** Descriptive characteristics of the study population. BMI = body mass index, BP = blood pressure.

Characteristics		All	Coughers n (%)	Controls n (%)	*P*-value
Age (Years)		51.2 ± 10.6	51.74 ± 11.36	50.81 ± 9.71	0.70
Sex	Male n (%)	39 (52.7)	18 (467.4)	21 (58.3)	0.23
	Female n (%)	35 (47.3)	20 (52.6)	15 (41.7)	
Smoking Status	Yes n (%)	17 (23)	12 (31.6)	5 (13.9)	0.06
	No n (%)	57 (77)	21 (68.4)	31 (86.1)	
BMI (kg/m2)		24.38 ± 6.79	24.35 ± 7.21	24.43 ± 6.42	0.94
BP	Mean SBP (mm Hg)	124.9 ± 9.34	125.92 ± 9.71	123.89 ± 8.9	0.57
	Mean DPB (mm Hg)	79.6 ± 7.41	79.74 ± 7.79	79.58 ± 7.11	0.56
Family History	Yes n (%)	41 (55.4)	20 (52.6)	21 (58.3)	0.39
	No n (%)	33 (44.6)	18 (47.4)	15 (41.7)	
ACE Inhibitor	Ramipril, n (%)	45 (60.9)	22 (57.9)	23 (63.9)	0.92
	Captopril, n (%)	15 (20.3)	8 (21.1)	7 (19.4)	
	Lisinopril, n (%)	6 (8.1)	4 (10.5)	2 (5.6)	
	Enalapril, n (%)	8 (10.8)	4 (10.5)	4 (11.1)	
Frequency of Cough	Once a week, n (%)	28 (37.8)	27 (71.1)		<0.001
	Twice a week, n (%)	5 (6.8)	5 (12.12)		
	3 to 5 times a week n (%)	5 (6.8)	5 (13.2)		

### Incidence of cough

Dry cough as an ACEI side effect was recorded systematically in the clinical records and defined as a cough that develops after the prescription of ACEIs in the absence of asthma or upper respiratory infection and whose symptoms disappear within 15 days of stopping the treatment. Cough caused by other conditions (acute RTI or other respiratory diseases) was not considered to be induced by ACEIs. Based on these criteria, patients were divided into the case group (coughers) and the control group (non-coughers). Of these 74 participants, the prevalence of cough related to ACEIs occurred in fifty-one percent (51%) patients, and these subjects were defined as case groups (coughers), while the other forty-eight percent (49%) individuals without ACEI-induced cough were classified as control group (non-cougher) (Table 1). There were 18 men and 20 women in the coughers group and 21 men and 15 women in the non-coughers group.

### Genotype frequencies & association with cough

Genotype frequencies of the various polymorphisms in the study sample are shown in Table 2. Of the 74 patients, 67 samples were sequenced representing 90.5% sequencing success, while seven samples could not be sequenced due to the contamination and low yield of DNA. A large amount of missing sequencing data adversely affects the power of analysis; however, we are missing less than 10% of data in our investigation which is likely to have a very small effect on the outcomes. For rs4149057, the genotype frequency of TT was 76% and 18% for TC in the cougher group while in the controls, TT was 75% and TC was 11% (p: 0.309, OR (95% CI): 2.08 (0.507,8.543)) (Table 3). There was no significant association of this SNP with ACE-Is-induced cough (Table 3). Even after adjusting for sex and smoking status, we could not find a significant association of this genetic variant with the cough. For rs2291075, the genotype frequency of CC was 55% and 40% for TC in the cougher group while in the controls, CC was 61% and TC was 25% (p: 0.231, OR (95% CI): 0.519 (0.178,1.517)). There was no significant association of this SNP with ACEI-induced cough either before or after adjusting for sex and smoking status.

**TABLE 2:** The following SNP markers have been identified in *SLCO1B1* gene in our hypertensive cohort.

S/No	References	Position	REF/ALT alleles	Consequence	Effect	Minor allele frequency (%)
1	rs4149057	12:21178665	T/C	synonymous variant	L191L	16.4
2	rs2291075	12:21178691	C/T	synonymous variant	F199F	35.8
3	rs556914358	12:21178695	A/G	missense variant	K201E	3
4	rs576844894	12:21178782	G/C	intron variant	Not Translated	4.5
5	rs201722521	12:21178926	A/G	missense variant	I211M	6
6	rs4149056	12:21178615	T/C	missense variant	V174A	41.8

**TABLE 3:** Association of *SLCO1B1* genetic polymorphisms with the risk of ACE-Is-induced cough. Homozygous wild-type patients served as the reference group. OR = odds ratio; CI = confidence interval. * Uncorrected P-value and crude OR using χ2 tests with Pearson 2 × 2 test or Fisher exact test. + Adjusted data by multivariate logistic regression analysis for sex, and smoking status.

	Coughers n (%)	Controls n (%)	Without adjustment*		With adjustment+	
			*p*-value	Or (95% CI)	*p*-value	Or (95% CI)
rs4149057						
TT	29 (76.3)	27 (75.0)				
TC	7 (18.40	4 (11.1)	0.309	2.08 (0.507,8.543)	0.309	0.480 (0.117,1.971)
rs2291075						
CC	21 (55.3)	22 (61.1)				
CT	15 (39.5)	9 (25.0)	0.231	0.519 (0.178,1.517)	0.231	1.925 (0.659,5.624)
rs556914358						
AA	35 (92.1)	30 (83.3)				
AG	1 (2.6)	1 (2.8)	0.903	1.196 (0.068,21.067)	0.903	0.836 (0.047,14.721)
rs4149056						
TT	12 (31.6)	11 (30.6)				
TC	15 (39.5)	13 (36.1)				
CC	07 (18.4)	09 (25.0)	0.556	1.714 (0.285,10.303)	0.556	0.583 (0.097,3.506)
rs576844894						
GG	35 (92.1)	29 (80.6)				
GC	1 (2.6)	2 (5.6)	0.531	0.446 (0.036,5.584)	0.531	2.242 (0.179,28.07)
rs201722521						
AA	34 (89.5)	29 (80.6)				
AG	2 (5.3)	2 (5.6)	0.865	1.196 (0.151,9.476)	0.865	0.836 (0.106,6.62)

Similarly, when genotype frequencies were calculated for rs556914358, AA was 92% and AG was 3% in the cougher group while in the controls, AA was 83% and AG was 3% (p: 0.903, OR (95% CI): 1.196 (0.068,21.067)). There was no significant association of this SNP with ACEI-induced cough. Genotype frequencies for SNPs rs4149056, rs576844894, and rs201722521 are provided in Table 3. Our analyses show no significant association with ACEIs-induced cough either before or after adjustment for sex and smoking status.

### Haplotype frequencies & association with cough

**FIGURE 1:**
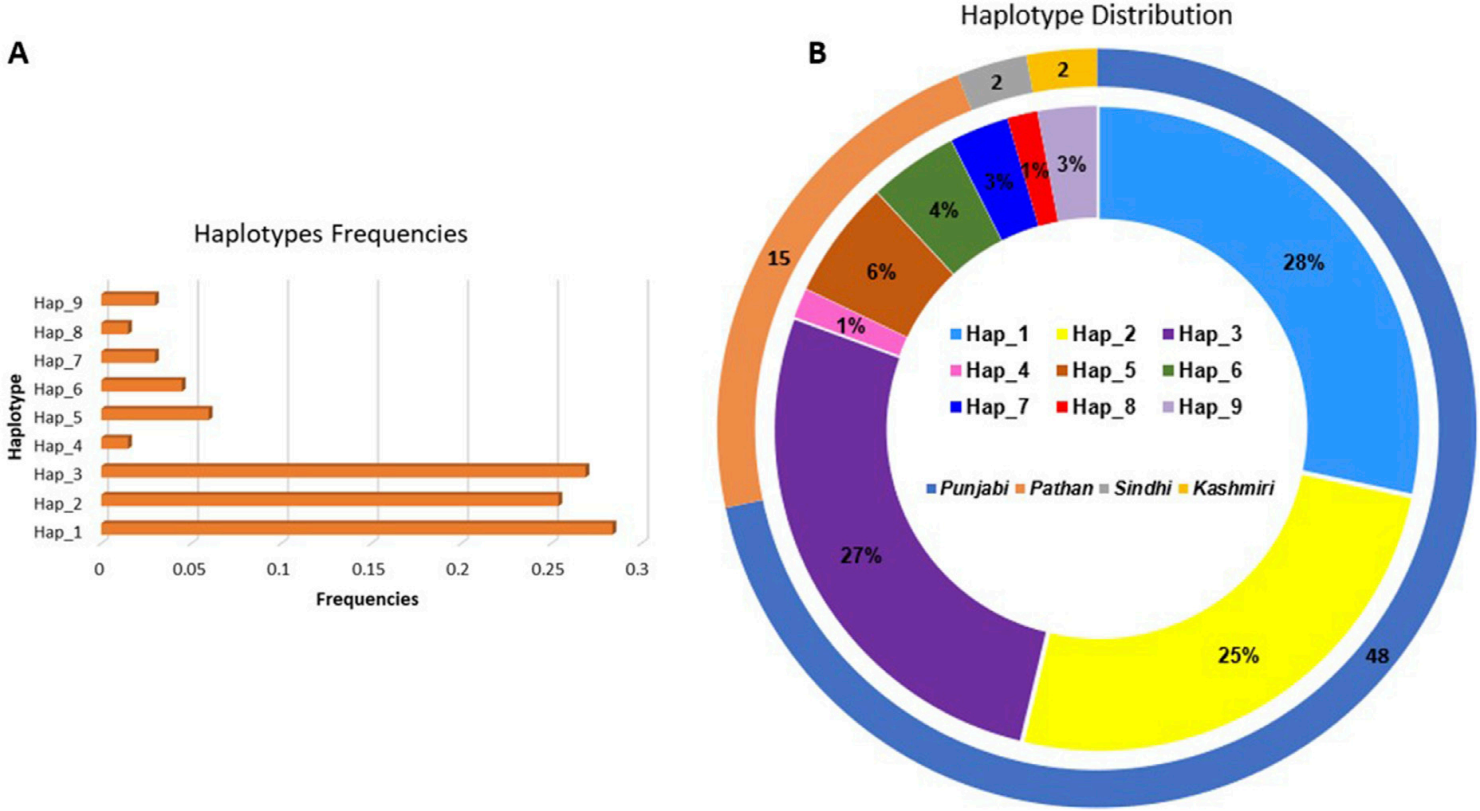
**(A)** Bar chart illustrating the frequencies of each haplotype observed in the dataset. The *x*-axis represents the haplotype frequencies, while the *y*-axis displays the individual haplotypes **(B)** Double-layered ideogram presenting the haplotype distribution across different ethnic groups. The outer ring (ideogram) showcases the distribution of sample sizes among four ethnic groups: Punjabi, Pathan, Sindhi, and Kashmiri, with the corresponding percentages indicated. The inner ring (ideogram) provides a detailed view of the distribution of the nine identified haplotypes within these ethnic groups, with each color-coded segment representing a specific haplotype.

Arlequin v3.5 calculated the frequency of each haplotype within the overall sample population. It tested for the significant differences in haplotype distribution between groups defined by phenotypic traits, such as the presence or absence of ACEI-induced cough. We found 9 haplotypes in our cohort (Figure 1; Table 4), 7 of which we investigated for their association with cough, the remaining 2 haplotypes were found in extremely low frequency. The frequency of haplotype H1 was 24% in coughers and 28% in controls, H2 was 29% in coughers and 17% in controls, H3 was 26% in coughers and 22% in controls. The frequency of haplotype H4 was 3% in coughers and 0% in controls and after adjusting for sex and smoking status showed a significant association with cough. H5 did not show a significant association with cough. Similarly, the frequency of haplotype H6 was 3% in coughers and 6% in controls. The frequency of haplotype H7 was 0.02 in coughers and 0.05 in controls while haplotype H9 was 3% in coughers and 3% in controls. Ultimately, out of nine haplotypes identified, only the haplotype (H4) shows significant accusation with the ACEI-induced cough. Other haplotypes did not exhibit significant association (Table 4).

**TABLE 4:** Association of haplotypes in *SLCO1B1* gene with the risk of ACE-Is-induced cough. Presence of a particular haplotype served as the reference group. OR = odds ratio; CI = confidence interval. * Uncorrected *P*-value and crude OR using χ2 tests with Pearson 2 × 2 test or Fisher exact test. + Adjusted data by multivariate logistic regression analysis for sex, and smoking status.

Haplotypes	Coughers n (%)	Controls n (%)	Without adjustment*		With adjustment+	
			*p*-value	Or (95% CI)	*p*-value	Or (95% CI)
H1						
Yes	9 (23.7)	10 (27.8)				
No	27 (71.1)	21 (58.3)	0.653	0.556 (0.043,7.214)	0.653	1.800 (0.139,23.374)
H2						
Yes	11 (28.9)	6 (16.7)				
No	25 (65.8)	25 (69.4)	0.327	0.273 (0.020,3.666)	0.327	3.667 (0.273,49.288)
H3						
Yes	10 (26.3)	8 (22.2)				
No	26 (68.4)	23 (63.9)	0.485	0.400 (0.030,5.248)	0.485	2.500 (0.191,32.802)
H4						
Yes	1 (2.6)	0 (0)				
No	35 (92.1)	31 (86.1)	1.000	0.00 (0.00,.)	0.000	21795213.934 (21795213.934, 21795213.934)
H5						
Yes	2 (5.3)	2 (5.6)				
No	34 (89.5)	29 (80.6)	0.661	0.500 (0.023,11.089)	0.661	2.000 (0.090,44.350)
H6						
Yes	1 (2.6)	2 (5.6)				
No	35 (92.1)	29 (80.6)	1.000	1.000 (0.034,29.807)	1.000	1.000 (0.034,29.807)
H7						
Yes	1 (2.6)	1 (2.8)				
No	35 (92.1)	30 (83.3)	0.711	0.500 (0.013,19.562)	0.711	2.000 (0.051,78.250)

## Discussion

Our study shows that Ramipril was the most frequently prescribed ACEI (60%), followed by Captopril (20%), enalapril (10.8%), and lisinopril (8%). We found the incidence of cough in the ACE inhibitor users to be 51%. We also found 6 SNPs and 9 haplotypes with varying frequencies in our cohort of hypertensive patients using ACE inhibitors. None of the SNPs and haplotypes was associated with ACE inhibitors-induced cough except haplotype 4 which was found significantly associated with cough after adjusting the sex and smoking status of the patients.

Globally, the incidence of ACEI-induced cough has been reported to be in the range of 20%–50% among patients treated with ACEIs and the onset of cough may be within days or weeks of beginning ACEI therapy (Simon et al., 1992). A significant delay between the onset of symptoms and actual diagnosis may predispose patients to unnecessary prescriptions and diagnostic tests. Therefore, awareness of, and early intervention for, this adverse event can result in significant cost savings and improvement in quality of life. According to our study, cough displayed the highest incidence (51%), followed by dizziness (18%), fainting (9%), and hypotension (8%). The incidence of ACEI-induced cough was higher in our study compared to some previous investigations carried out in Pakistan. One study found the frequency of dry cough with ACE inhibitors around 17%, ranging from almost 20% with Enalopril, 16.6% with Captopril, 10% with Lisinopril and Ramipril, 15% with Qurinapril and Perindopril (Saboor et al., 2012). Another study reported a slightly higher incidence rate of dry cough with ACEI. Jamshed and colleagues reported a 26.8% incidence of dry cough with ACE inhibitors. They found that Enalapril was the most commonly ACEI prescribed among patients with ACEI-induced cough (Jamshed et al., 2019). Other studies reported similar frequencies of ACE inhibitor-induced cough in the Malaysian and Chinse populations (24.1% and 32% respectively) (Loo et al., 2022; Luo et al., 2015). One reason for a slightly lower incidence of dry cough in these studies might be the fact that our study included mild cases of dry cough also, while other investigators, for example, Jamshed et al., included only those cases in which dry cough was severe enough for the patients where they were contemplating requesting physician to change the drug.

Our study did not find a significant association of any SNP with cough except for the haplotype H4 which was significantly associated with cough after adjusting for sex and the smoking status. After adjustment, patients with H4 were 22 times more likely to have the ACE inhibitor-induced cough. While there are other confounding factors such as age, co-morbidities, and concurrent medications, and they all are important to consider, we only adjusted for sex and smoking status as per the practice of previous studies (Luo et al., 2015). Literature reveals several studies showing the association of genetic variants in various genes with ACEI-induced cough. Luo and colleagues investigated *ABO* genetic polymorphisms rs8176740 and rs495828 for their possible association with ACEI-induced cough. They found rs495828 polymorphism was associated with enalapril-induced cough in Chinese patients with essential hypertension (Luo et al., 2014). In another study, Kim et al., detected two SNPs in the *NK2R* gene (i.e., Gly231-Glu and Arg375His), and found that the Gly231Glu polymorphism was associated with a lower prevalence of ACEI-related cough (Kim et al., 2009).

In previous studies, the genetic variants of *SLCO1B1* were proven to be relevant to the response to drugs transported by OATP1B1, such as statin-induced myopathy, irinotecan-induced toxicities, methotrexate clearance (Carr et al., 2013; Link et al., 2008; Hussain et al., 2023). The *SLCO1B1* 521T > C polymorphism appeared consistently relevant, but results regarding the *in-vitro* association of the 597 C>T variant, which may make the transport activity increase, decrease, or not change, were conflicting (Niemi et al., 2011). In China, the 521T > C variant was reported to be an important determinant of the PK of ACEIs (Tian et al., 2011) while in the Russian population, dry cough attributed to enalapril was associated with polymorphisms rs2306283 in the *SLCO1B1* gene (Sychev et al., 2023) However, in our investigation, we did not observe a significant association. In another investigation, Luo and colleagues provided evidence that the *SLCO1B1* functional genetic variant (521T > C; Val174Ala) substantially alters the risk of enalapril-induced cough in the Chinese population (Luo et al., 2015). No significant association with this genetic variant in our study might be due to the underlying genetics of our population or it may be simply due to the smaller sample size of our cohort. In addition to population-specific differences and low sample size, another possible reason for not finding significant associations in our study might be the potential environmental factors such as diet, temperature, oxygen levels, humidity, light cycles, and the presence of mutagens, which may also be responsible for obscuring the potential association with ACE inhibitor-induced cough.

New determinants of ACEI toxicity are also being investigated. Ghouse and colleagues found seven risk loci for ACEI discontinuation (Ghouse et al., 2022). Their analysis showed several pathways involved in ACEI-associated cough as the main underlying phenotype. They specifically identified new loci including rs7526729 in *KCNA2*, rs1544730 in *SRBD1,* rs16870989 in *KCNIP4,* rs12210271 in *PREP*, rs360206 in *SCAI*, rs8097200 in *L3MBTL4,* and rs6062847 in *SLCO4A1/NTSR1*associated with ACEI discontinuation. Such studies emphasize the relevance of utilizing proxies for ACEI ADRs to provide important insights into the etiology and genetic architecture of specific adverse drug reactions. In this context, our findings related to the association of haplotype H4 in the *SLCO1B1* gene with cough may prove important for predicting ACEI-induced cough in prospective patients.

The findings of our study that there exists a significant association between genetic variation in the *SLCO1B1*, particularly the H4 haplotype, and cough signifies that H4 is likely to increase the risk of developing cough in hypertensive patients taking ACE inhibitors. H4 may also modify the intake of statins to hepatocytes from the blood. Therefore, the observed association between *SLCO1B1* genetic variation and ACE inhibitors-induced cough may result from the pharmacokinetic mechanism, which ultimately controls the plasma levels of ACE inhibitors. Thus, *SLCO1B1* may be a suitable gene to identify hypertensive patients on ACE inhibitors who are more likely to suffer from ACE inhibitor-induced cough. It has been suggested previously that genotyping SLCO1B1 variants might be a useful strategy to achieve the benefits of enalapril treatment more effectively and safely in China (Luo et al., 2015). These observations will help provide pharmacogenetic markers for ACE inhibitors-based treatments. However, larger trials with higher sample sizes, especially those with functional studies to further investigate the underlying biological mechanisms are required to shed more light on the findings of the current study.

While the findings of this study are important and valid, we identify several shortcomings in our investigation. While the partial gene sequencing approach has previously yielded good results (Ahmed et al., 2022), it nevertheless, misses out on significant pieces of information by not sequencing the whole gene. Furthermore, a low sample size in our study also precludes a strong generalization of the study findings. This is perhaps why we could not find more SNPs and haplotypes strongly associated with the incidence of cough in our study.

## Conclusion

Our study shows that the incidence of ACE inhibitors-induced cough is very high, compared to other populations, in the Pakistani hypertensive cohort investigated in this study. We also found that genetic variants of *SLCO1B1* genes may be associated with the cough induced by ACEIs. Although *SLCO1B1* 521T > C polymorphism was not significantly associated with cough, specifically haplotype, H4 was found significantly associated with cough after adjusting for sex and smoking status. These findings emphasize the significance of *SLCO1B1* genetic factors as potential predictors of ACEI-induced cough. Our research on the pharmacogenomics of the relationship between *SLCO1B1* genetic variants and ACEI-induced cough may contribute to the therapeutic use of *SLCO1B1* pharmacogenetics for predicting ACEI-induced cough.

## Data Availability

The data supporting the findings of this study have been deposited in GenBank under the accession number [PP387529-PP387595].
